# Estimating blood pressure from the electrocardiogram: findings of a large-scale negative results study

**DOI:** 10.1088/1361-6579/ae1926

**Published:** 2025-11-10

**Authors:** Seyedeh Somayyeh Mousavi, Sajjad Karimi, Mohammadsina Hassannia, Zuzana Koscova, Ali Bahrami Rad, David Albert, Gari D Clifford, Reza Sameni

**Affiliations:** 1 Department of Biomedical Informatics, Emory University School of Medicine, Atlanta, GA, United States of America; 2 AliveCor Inc., Mountain View, CA, United States of America; 3 Department of Biomedical Engineering, Georgia Institute of Technology, Atlanta, GA, United States of America

**Keywords:** electrocardiogram, blood pressure, machine learning, estimation, classification, demographics, explainable AI

## Abstract

*Objective.* Electrocardiography and blood pressure (BP) measurement are two widely used tools for diagnosis and monitoring cardiovascular diseases. While the electrocardiogram (ECG) and BP have been considered complementary modalities, there are also systematic relationships between them. Therefore, advancements in portable and wearable ECG devices, along with promising results in cuff-less BP measurement using a combination of ECG and other bio-signals have led researchers to hypothesize the possibility of estimating BP and classifying BP categories (e.g. normal vs. hypertensive) using only ECG. However, the literature is divided on this topic: some studies support this hypothesis, while others reject it. *Approach.* In this study, regression and classification machine learning (ML) models were developed to explore the feasibility of estimating BP and predicting BP categories (normal vs. hypertensive) from 30 s ECGs using an extensive dataset from AliveCor Inc. which includes 124 427 records from 7412 subjects. The ECG and BP recordings were asynchronous with variable counts and time lags. Therefore, a 3.5 min time window before and after each ECG recording was used to calculate the mean BP measurement. Sex-aware ML models were trained using a comprehensive feature vector comprising 280 features: 128 explainable ECG features developed by the research team and 150 ECG features extracted by the Black Swan team, one of the top-performing teams in the PhysioNet Challenge 2017. Additionally, the average time gap between each ECG and the corresponding BP measurement, along with the subject’s age, were included as two supplementary features. *Main results.* Our best regression ML models achieved a mean absolute error of 12.59 mmHg for estimating systolic BP and 7.43 mmHg for diastolic BP, with correlation coefficients of 0.35 and 0.38 between the predicted and actual values, respectively. The best BP normal-hypertensive classification model achieved an area under the receiver operating characteristic curve of 0.655. *Significance.* Using a large dataset of ECG and BP recordings, this study found that ML models did not achieve acceptable performance in predicting BP values or classifying BP categories, indicating that BP cannot be reliably estimated from the ECG.

## 1. Introduction

Blood pressure (BP) measurement and electrocardiography are two complementary methods widely used for cardiovascular monitoring and diagnosis. BP is influenced by cardiac mechanical function and systemic vascular resistance (Mousavi *et al* 2024). When the heart contracts, it creates a pulsatile pressure wave in the arterial system (Nichols *et al* 2022). The systolic blood pressure (SBP) represents the maximum and the diastolic blood pressure (DBP) reflects the minimum of the pressure wave in each cardiac cycle, both of which are time-varying due to natural fluctuations and measurement errors and biases, such as those caused by respiration (Pickering *et al* 2005, Mousavi *et al* 2024, Mukkamala *et al* 2025). Both tonic and cyclic fluctuations in the BP wave provide critical insights into cardiovascular health, making BP monitoring a standard in patient care and an effective tool for cardiovascular diseases (CVDs) diagnosis and management (Muntner *et al* 2019, Mousavi *et al* 2024). The guidelines for hypertension recommend that symptomatic individuals regularly monitor their BP (Reboussin *et al* 2018).

On the other hand, the electrocardiogram (ECG) measures the electrical function of the heart and captures the electro-physiological patterns of depolarization and re-polarization during each cardiac cycle (De Luna *et al* 2006, Kaplan Berkaya *et al* 2018). ECG recording is cost-effective, accurate and commonly available in most health centers and outside clinical settings using portable and wearable ECG monitors, making it suitable for long-term cardiac monitoring and CVD detection (Shah *et al* 2021, Neri *et al* 2023, Muzammil *et al* 2024).

Previous studies have attempted to estimate BP using machine learning (ML) or deep learning (DL) methods from only photoplethysmography (PPG) signals (Mousavi *et al* 2019, Ma *et al* 2023, Apple Inc. 2025), or from a combination of PPG with other biosignals such as the output signal of a Hall sensor (Lee *et al* 2011, Nam *et al* 2013), the modulated magnetic signature of the blood (Zhang *et al* 2016), ballistocardiography (Chen *et al* 2013, Kim *et al* 2018), and impedance plethysmography (Liu *et al* 2017, Huynh *et al* 2019). Researchers have further hypothesized the feasibility of estimating BP using only ECGs and electro-physiological features (Mousavi *et al* 2019, 2020, Bird *et al* 2020, Sato 2021, Landry and Mukkamala 2023). Rapid advancements in home care devices, such as portable ECG devices, smartwatches, and smartphones, have further motivated efforts to integrate BP measurement functionality into these technologies (Shah *et al* 2021). However, the literature is divided on whether BP can be accurately estimated from ECGs: those whose results support this hypothesis and those that do not.

Methodologically, the literature on the relationship between ECG and BP has addressed two main problems: (1) estimating BP values, and (2) predicting BP categories (e.g. normal vs. hypertensive cases) using simultaneous or recent ECG records (Angelaki *et al* 2022, 2024, Liang *et al* 2024). Technically, the first problem requires a regression-based ML framework, while the second is a classification task. In this study, we primarily focus on the BP prediction problem as our main objective, but we also present BP category estimation results on the same dataset to enable comparisons in future research.

In 2008, Ali Hassan *et al* (2008) developed a linear regression model to estimate SBP from heart rate (HR) extracted from 30 s ECG recordings of 10 normal-ECG subjects. BP values were also measured manually. For each individual, 20 records were used to develop the regression model and the 10 remaining ones were used for testing. To generalize SBP estimation for new subjects, the final model slope was obtained by averaging the individual regression slopes across all participants.

In 2018, Simjanoska *et al* (2018) developed an ML model for estimating BP using ECGs. The study analyzed 3129 ECGs with a length of 30 s, from 51 subjects, including both healthy and unhealthy individuals. The feature vector consisted of seven components: signal mobility, signal complexity, fractal dimension, entropy, autocorrelation, age, and hypertension classification. Four models were developed: one classification model to predict the hypertension group, and three regression models to estimate SBP, DBP, and mean arterial pressure (MAP). The mean absolute error (MAE) and standard deviation (SD) of the regression model were 7.72±10.22 mmHg for SBP and 9.45±10.03 mmHg for DBP. The group further extended their study by employing a different pre-processing approach, adjusting the cutoff frequency of filters, and utilizing ECGs with different lengths of 10, 20, and 30 s (Simjanoska *et al* 2020). The results showed an MAE and SD of 16.60±11.05 and 9.24±7.85 mmHg for SBP and DBP, respectively.

In 2020, Miao *et al* (2020) developed a DL model for estimating BP from ECG, utilizing a residual network and long short-term memory, to capture both time and spatial information from ECGs. The model was trained and tested on the public Multiparameter Intelligent Monitoring in Intensive Care (MIMIC-III) database, which includes ECG and invasive BP information from individuals in critical care units (Johnson *et al* 2016). The pre-processed dataset consisted of 1711 subjects and 897 743 records, each with a length of 2.5 s. The developed approach achieved a ME with a SD of $-0.22\pm$5.82 mmHg for SBP and $-0.75\pm$5.62 mmHg for DBP. The correlation coefficients between the estimated and actual values were 0.88 and 0.71 for SBP and DBP, respectively. Table 1 presents the results of studies based on ML and DL approaches for estimating BP using ECGs.

**Table 1.** Comparison of studies using machine learning and deep learning (DL) models for blood pressure estimation based solely on ECG data.

	Data				SBP (mmHg)					DBP (mmHg)				
Study	# Records	# Subjects	ECG len.	# Features	ME	STD	MAE	STD	*ρ*	ME	STD	MAE	STD	*ρ*
(Simjanoska *et al* 2018) 2018	3129	51	30 s	7	—	—	7.7	10.2	—	—	—	9.5	10.0	—
(Mousavi *et al* 2018) 2018	660	220	15 s	—	—	—	12.8	12.2	—	—	—	6.0	6.4	—
(Simjanoska *et al* 2020) 2020	3129	51	30/20/10 s	7	—	—	16.6	11.1	—	—	—	9.2	7.9	—
(Banerjee *et al* 2022) 2022	2000	—	16 s	—	0.8	9.1	6.0	—	—	0.1	6.2	3.5	—	—
(Wuerich *et al* 2022) 2022	—	942	4 s	34	5.9	7.2	0.9	—	—	—	—	3.7	5.2	0.92
(Syah *et al* 2023) 2023	56	—	20 s	3	—	—	2.8	1.5	0.95	—	—	2.9	2.1	0.93
(Aldein *et al* 2023) 2023	4904	—	32 s	—	—	—	6	4.5	—	—	—	2.5	3.7	—
(Kuzmanov *et al* 2024) 2024	130 461	—	30 s	—	—	—	10.9	—	—	—	—	6.6	—	—
(Aldein *et al* 2025) 2025	12 000	942	32 s	—	—	—	5.50	4.28	—	—	—	2.50	3.80	—
(Miao *et al* 2020) 2020	897 743	1711	2.5 s	DL	–0.1	10.0	7.1	—	0.88	0.0	6.3	4.6	—	0.71
(Fan *et al* 2021) 2021	21 422	—	10 s	DL	0.1	10.8	7.7	—	—	0.1	5.9	4.4	—	—
(Fan *et al* 2020) 2021	21 422	—	10 s	DL	0.2	10.8	7.2	—	—	1.2	5.9	3.9	—	—

At the same time, some research has questioned the feasibility of ECG-based BP estimation. Sato *et al* (2021) and Landry and Mukkamala (2023) are two studies in this category, which based on the electrophysiology of BP and ECG and the shortcomings in the reported results in the literature, have debated that accurate ECG-based BP estimation is unfeasible. They have not conducted any independent experiments to support this claim.

This study aims to explore the feasibility of estimating BP using only ECGs using ML models trained on a large ambulatory dataset, while addressing shortcomings in former methodologies. A comprehensive set of 278 engineered features, derived from the time, frequency, and time–frequency domains of the ECGs, and used as inputs to regression models for BP estimation. The models are designed to be demographic-aware by incorporating the sex and age of subjects, which are known to significantly influence BP values (Mousavi *et al* 2024). All ECGs are standardized to a fixed length of 30 s to ensure consistency across records. Detailed data cleaning, sub-sampling, and standard cross-validation techniques are used to ensure that the results are not biased. *Our findings most strongly support studies that have concluded accurate ECG-based BP estimation is unfeasible.*

## 2. Method

### 2.1. Database

The data used in this study consists of ECG and BP measurements from two databases collected over two years from August 2019 to March 2021 by AliveCor (Mountain View, CA, USA), using the following devices:

(i) OMRON Complete (Omron Healthcare, Kyoto, Japan), which is an integrated BP monitor and single-lead ECG;
(ii) KardiaMobile (AliveCor, Mountain View, CA, USA) for collecting single-lead ECGs and independent BP readings from portable BP devices (Omron Healthcare, Kyoto, Japan).

To note, the ECG and BP were self-recorded asynchronously in non-clinical settings, with variable numbers of BP and ECG per subject and varying time gaps between the two modalities (varying between seconds and hours). The ECG dataset comprises 180 790 records from 10 624 subjects, with a minimum time gap of 30 seconds between two consecutive ECG recordings for each unique subject. ECGs were recorded at a sampling frequency of 300 Hz. The BP dataset consists of 21 227 729 measurements, corresponding to 297 965 subjects. A total of 10 346 subjects, which were common between the ECG and BP datasets, were shortlisted for this study.

### 2.2. Data cleaning

**Figure 1.**
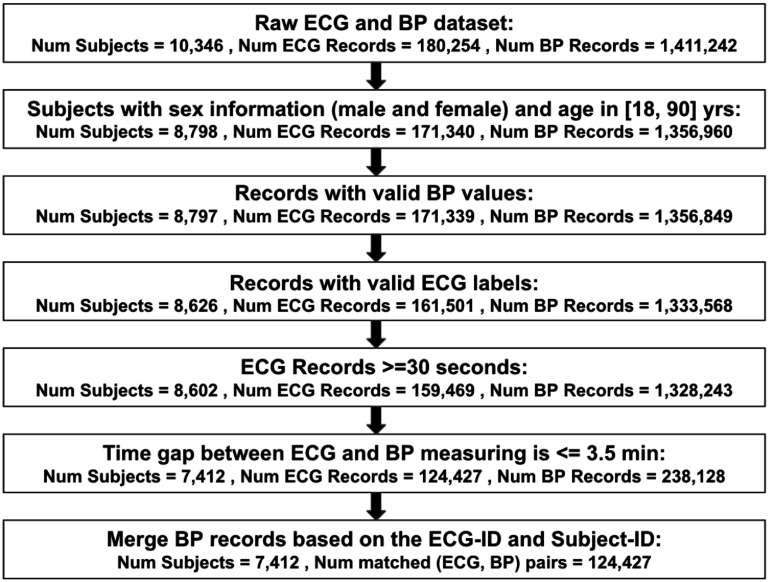
Data cleaning and workflow process for developing machine learning models to estimate blood pressure using only ECGs. Abbreviations: valid label:  ‘sinus rhythm’, ‘atrial fibrillation’, ‘bradycardia’, ‘tachycardia’, ‘too short’, ‘unclassified’, invalid label: ‘unreadable’ and missing values. Additionally, in the final stage up to 6 records per subject were selected, based on the median number of records per subject after excluding an outlier with 3500 records and subjects with only one record.

The data cleaning process is summarized in figure 1. Accordingly, records were selected from the matched dataset based on the following criteria:

(i) The analysis was limited to adult male and female subjects aged between 18 and 90 years at the time of ECG recording. Subjects with unknown sex or with age outside $[18,90]$ were excluded from the analysis.
(ii) Records with misreported DBP values higher than SBP were removed. Then, thresholds were applied to define valid BP ranges. Valid BP ranges were set to DBP between 20–200 mmHg and SBP between 30–300 mmHg. These thresholds are consistent with the pre-processing approach used in our previous study, which analyzed approximately 75 million BP values from the general population (Mousavi *et al* 2024).
(iii) The dataset included ECG classification labels generated by AliveCor’s proprietary ECG analysis software, which labels signals as ‘sinus rhythm’, ‘atrial fibrillation’, ‘bradycardia’, ‘tachycardia’, ‘unclassified’, ‘too short’, ‘unreadable’ and missing values. Records labeled as ‘unreadable’ or with missing labels were removed.
(iv) For consistency, ECG record lengths were fixed to 30 s, and the records shorter than 30 s were excluded from the analysis. Previous studies indicate that this duration is sufficient for capturing essential ECG features, especially for rhythm analysis and heart rate variability (HRV) (Munoz *et al* 2015). 98% of the ECG database complied with this requirement. For consistency, the ECGs longer than 30 s were truncated to the first 30 s.
(v) The ECG and BP data were collected asynchronously, resulting in varying time gaps between the ECG and BP measurements of the same subject. Given that both signals naturally fluctuate over time, we defined a maximum allowable time gap such that BP variability within this window would be minimal—ensuring that estimating BP from ECG remained both meaningful and clinically relevant. To determine this threshold objectively, we referred to acceptable BP error margins from BP device standards and reported rates of short-term BP variability in the literature. Presumably, as long as the time-gap between ECG and BP collection is within these thresholds, any BP change during that interval would fall within an acceptable error margin—making the ECG-BP pairing valid for estimation purposes.
  According to the Association for the Advancement of Medical Instrumentation (AAMI) standard, the mean BP error in BP measurement devices should be less than 5 mmHg (Stergiou *et al* 2018). To identify the time window during which a 5 mmHg change in BP might occur, relevant literature was reviewed. Most studies reported mean BP differences over 30 min or longer intervals (Mancia *et al* 1983, Graham *et al* 1995, Clement *et al* 2003, Kario *et al* 2003, Okamoto *et al* 2009, Sayk *et al* 2010, Mancia 2012). From these studies, reported mean BP differences and their corresponding time windows were extracted to estimate the ‘rate of BP variation’ over time. Using these rates, the time intervals corresponding to the negligible 5 mmHg change were calculated by dividing 5 mmHg by the rate of change. The resulting estimates ranged from 3.5 to 38 min. The minimum value (3.5 min), was considered as the acceptable short-term window, which we considered as the maximum allowable time gap between ECG and BP recordings.
(vi) Many subjects had multiple BP measurements within the acceptable BP-ECG time interval window. For each subject and ECG, all BP measurements within the acceptable time window of (3.5 min) were averaged. Averaging BP measurements within short time windows is a standard procedure in clinical practice, which results in more accurate BP measurements (Mousavi *et al* 2024), and reduction of measurement biases (Nateghi and Sameni 2025).

**Figure 2.**
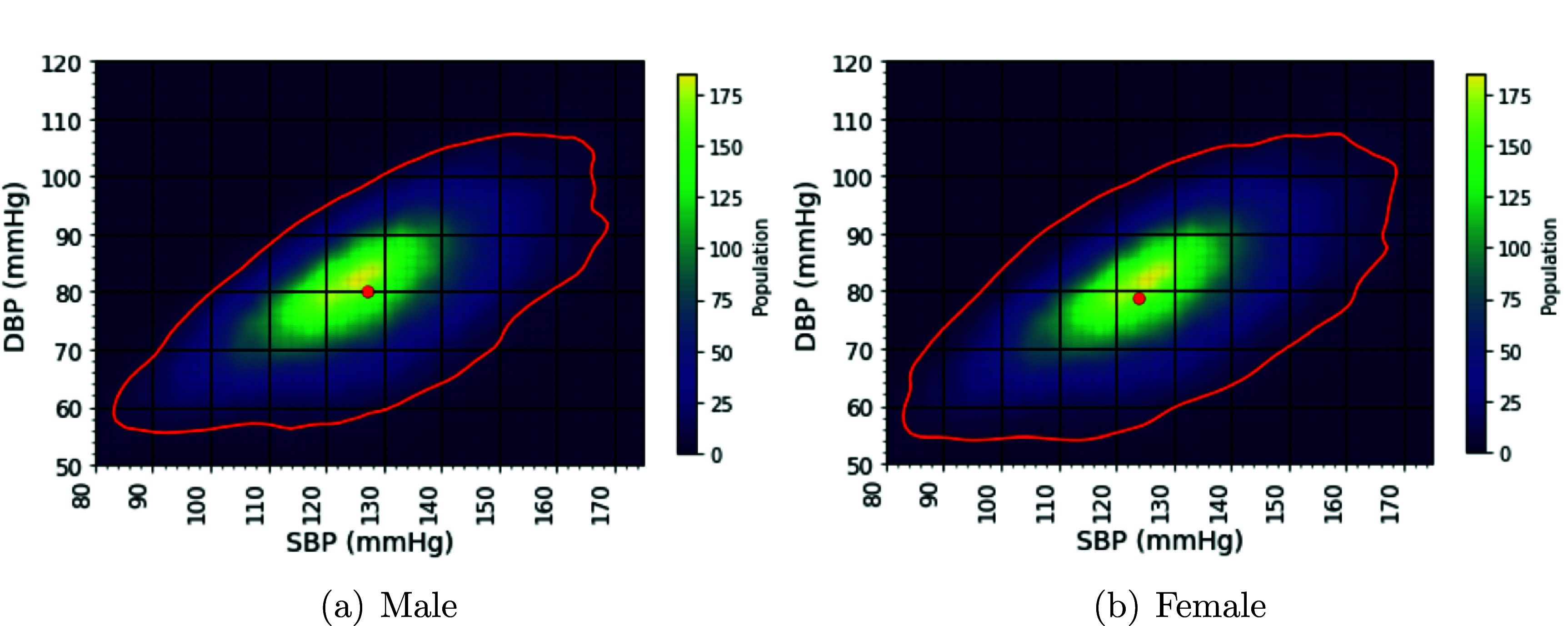
Comparisons of blood pressure distributions between sexes in the pre-processed data presented through heatmaps and contour plots, representing the 95% percentiles of the BP values within the contours. Dots indicate the mean SBP and DBP values. Mean SBP values are 127.1 and 123.8 mmHg, and mean DBP values are 80.2 and 78.9 mmHg for male and female subjects, respectively.

 The final filtered dataset included 124 427 pairs of BP and ECG records from 7412 subjects. Table 2 summarizes the distribution of the final dataset by sex and ECG labels, where ‘Normal’ represents ‘sinus rhythm,’ while all other labels including ‘atrial fibrillation’, ‘bradycardia’, ‘tachycardia’, and ‘unclassified’ are considered ‘Abnormal’. Also, table 3 presents the statistical distribution of BP datasets by sex and ECG labels, and figure 2 illustrates the 95% percentile range contours for males and females BP distribution. The mean SBP and DBP for each group are marked with dots.

**Table 2.** Distribution of the final processed ECG records based on sex and label.

Sex	# Total	# Abnormal	# Normal
Female	23 597	2846	20 751
Male	100 830	14 462	86 368

**Table 3.** Minimum, maximum, mean, and standard deviation of systolic and diastolic blood pressure (SBP and DBP, in mmHg) of the filtered dataset, categorized by sex and ECG label, for all records and those with sinus rhythm (normal).

Sex	BP type	ECG label	Min	Max	Mean	STD
Male	SBP	All	60	227	127.1	16.0
Male	SBP	Normal	65	227	127.3	15.8
Male	DBP	All	40	165	80.2	10.5
Male	DBP	Normal	40	165	80.1	10.2
Female	SBP	All	61	215	123.8	17.0
Female	SBP	Normal	62	215	124.0	16.7
Female	DBP	All	40	147	78.9	11.0
Female	DBP	Normal	40	147	78.8	10.8

### 2.3. ECG pre-processing

The ECG records were band-pass filtered with a band-pass frequency between 0.1 Hz and 100 Hz, and a notch filter at 50 or 60 Hz, depending on the local power line frequency. The notch filter was designed using a second-order infinite impulse response filter (iirnotch in MATLAB) with a quality factor (*Q*) of 40 and was applied.

### 2.4. Feature extraction

A total of 280 features were extracted, comprising 128 interpretable features extracted from the ECG records using a codebase developed by our team (Sameni 2006–2025); 150 features extracted using the Black Swan codebase (Zabihi *et al* 2017); the time gap between the ECG and the average time of the corresponding BP measurements (within 3.5 min time windows); and the subject age. To enable the replication of the implemented process, the complete feature set is described below.

(i) *Beat signal-to-noise ratio (SNR)*: To quantify beat-to-beat morphological consistency in the ECG over the 30 s segment, a SNR index was computed and assigned to each beat. R-peaks were first detected using the OSET robust R-peak detector function peak_det_likelihood (Sameni 2006–2025), and individual beats were segmented using a window of *W* samples centered around each R-peak. Robust weighted average (RWA) and robust beat median (RBM) beats were then calculated, following the method in Leski (2002). For each beat, the residual was computed as the difference from the RWA or RBM beat, and the beat SNR was defined as the power ratio between the original beat and the mean/median-based residuals. These SNRs capture both morphological deviations and measurement noise.
(ii) *HRV and HR metrics:* After ECG R-peak detection, R-R intervals were computed and converted to instantaneous HR values in beats per minute (bpm). The HR sequence was next summarized using the mean, median, 5th percentile, and 95th percentile. HRV was assessed using the standard deviation of R-R intervals and the root mean square of successive differences (Clifford *et al* 2006).
(iii) *Time interval measurements*: Fiducial points for each beat were extracted using the fiducial_det_lsim function from OSET (Sameni 2006–2025). Using these points, key ECG time intervals were calculated, including the QRS complex duration, QT interval, PR interval, ST interval, PR segment level, and ST segment level. Additional intervals were computed between specific peak pairs: P–R, Q–R, S–R, and T–R, to capture more detailed temporal relationships between waveform components. Corrected QT intervals (QTc) were also derived using the Bazett (QTc-B) and Fridericia (QTc-F) corrections (Luo *et al* 2004).
(iv) *Amplitude and morphological area metrics*: Amplitude and area-based features were computed using fiducial points marking the onset, peak, and offset of each ECG waveform component. For each component, the amplitude and the area under the curve (sum of ECG values from onset to offset) were calculated. In addition, we computed the amplitude ratio of the R peak to other major peaks (P, Q, S, and T), and the amplitude difference between the S and T peaks across the ST segment.
(v) *Amplitude-to-timing ratios*: For each beat, the difference between the R-peak amplitude and the amplitude of other peaks was divided by the time interval between the R peak and the corresponding peak, providing a measure of waveform shape (slope).
(vi) *Signal mobility and complexity*: *Mobility* was computed as the ratio of the variance of the first derivative of the ECG to the variance of the ECG (Simjanoska *et al* 2018, 2020, Fuadah and Lim 2022). *Complexity* was calculated as the ratio of the variance of the second derivative to the variance of the first derivative, divided by the mobility value (Simjanoska *et al* 2018, 2020, Fuadah and Lim 2022).
(vii) *Singular value decomposition (SVD) metrics*: SVD has been shown to encode ECG beat variability (Zheng *et al* 2021). ECG beats were segmented around each R-peak with a window of the median beat-to-beat interval, and stacked to form a 2D matrix (number of beats times number of samples of the segmented beats) using the event_stacker function from OSET (Sameni 2006–2025), where each row represents one beat. SVD was then applied to this matrix to extract singular values. The resulting values were normalized by the largest singular value and used as features to capture the similarity and reproducibility of ECG beats across the segment. The number of non-zero singular values of a rectangular matrix is smaller than or equal to the minimum of its rows and columns, which in our case was the number of beats used to construct the stacked beat matrix. To ensure a fixed feature length across all subjects and records, the SVD-based feature vector was set to a length of 45, corresponding to the maximum number of beats over 30 s across all subjects. Shorter vectors were zero-padded to reach this length.
(viii) *Black-Swan:* This set includes 150 features developed by a top-performing team in the PhysioNet Challenge 2017 for atrial fibrillation classification (Zabihi *et al* 2017). The features span multiple domains, including time, frequency, time-frequency, phase space, and meta-level representations. This set has also been successfully applied in other ECG classification tasks (Bahrami Rad *et al* 2021, 2024, Koscova *et al* 2024).

The amplitude, interval, and morphological features described above were computed per beat. These beat-wise values were then summarized using the mean, median, and SD to form fixed-length feature vectors.

### 2.5. ML models

Decision tree-based regression models were used for their performance, their ability to handle feature sets with missing values, and their capacity to model complex and nonlinear relationships in data (Podgorelec *et al* 2002). This includes extreme gradient boosting, random forest (RF), CatBoost, and light gradient boosting machine (LightGBM).

### 2.6. Model developing and data splitting

Our previous studies have shown that, at the population level, males exhibit higher BP than females (Mousavi *et al* 2024). Therefore, our SBP and DBP estimation models were trained separately for each sex group. Furthermore, for each sex group, two distinct BP models were trained using: (1) only normal-labeled ECG records and (2) all records. This allowed us to investigate whether BP estimation performance differs when trained exclusively on normal ECGs versus both normal and abnormal cases. As a result, four distinct models were developed for each of SBP and DBP (male-normal/all and female-normal/all). See table 3 for the breakdown.

For training and validation, we used *subject-level* data splitting rather than record-level to avoid inter-subject data leakage between training and validation, ensuring that the results are generalizable to other datasets. Accordingly, all models were trained using leave-subject-out five-fold cross-validation. The preprocessed dataset (detailed in figure 1) was randomly split into five sets of subjects. In each fold, the model was trained on data from four sets and tested on the left-out set. In this cross validation scheme, each subject appeared only once in the test set and four times in the training set (i.e. in 4/5 folds). In terms of ECG-BP recording pairs, the subjects had varied numbers of recordings: over 26% of the subjects had only one pair; the median was six pairs per subject; and, in an extreme case, one subject had 3689 measurement pairs. To address this imbalance and reduce the risk of biasing the ML models toward subjects with more measurements, the number of ECG-BP pairs per subject was capped at six in the train and test datasets. For subjects with more than six recordings, six pairs were randomly selected during cross-validation to make the best use of the available data. For subjects having six or fewer ECG-BP pairs, the same pairs were used for training across all folds. Therefore, most of the 124 427 pairs of ECG-BP measurements listed in figure 1 eventually contributed to the training/testing procedure.

In each fold, the model predictions were stored and after completing all five folds for each model, the predictions from all folds were aggregated to calculate and report the performance metrics. The training and test sets used for training each fold were identical across all the studied regression/classification models to accomplish a fair comparison.

### 2.7. Evaluation metrics

The performance of the developed ML models was evaluated using various metrics, including mean error (ME), SD of ME, MAE, SD of MAE, and correlation coefficient, to enable comparison with other studies. Specifically, the correlation coefficient reflects the strength of the linear relationship between the estimated and actual BP values. The correlation coefficient can be either positive or negative, implying a direct or inverse relationship. The absolute correlation coefficient ranges from 0, indicating no linear relationship, to 1, indicating a perfect relationship (Martin Bland and Altman 1986).

## 3. Results

### 3.1. ECG-based BP estimation

Table 4 summarizes the performance of the ML models in estimating DBP and SBP using only ECGs, based on sex and ECG labels (across all and normal-only ECG records). The best results, based on the correlation coefficient metric, were achieved in estimating DBP with a value of 0.38 using CatBoost and normal-ECG records of males, and in estimating SBP with a value of 0.35 using RF and all-ECG records of females.

**Table 4.** Performance of regression models (random forest (RF), CatBoost, and light gradient boosting machine (LightGBM)) for estimating systolic (SBP) and diastolic (DBP) blood pressure using 30 s ECGs with a 280-feature set, split by ECG labels and subject sex. The models were trained and validated using **leave-subject-out cross validation** (compare and contrast with record-wise dataset partitioning in table 6). The number of records per subject was limited to a maximum of six (median of the number of ECG-BP pairs per subject) to avoid biasing results by over-representing individuals with more data.

Data				SBP (mmHg)						DBP (mmHg)					
Label	Sex	# Subjects	# Test-records	MAE	STD	ME	STD	*ρ*	Model	MAE	STD	ME	STD	*ρ*	Model
All	Male	5236	20 107	11.82	9.69	0.03	15.29	0.29	LightGBM	7.62	6.24	0.04	9.85	0.37	LightGBM
All	Female	2176	7364	12.59	10.22	0.25	16.21	**0.35**	RF	8.03	6.33	0.19	10.23	0.38	RF
Normal	Male	4982	18 678	11.64	9.49	0.32	15.01	0.30	RF	7.43	6.08	0.03	9.60	**0.38**	CatBoost
Normal	Female	2078	6868	12.56	10.07	0.35	16.09	0.34	RF	7.90	6.18	0.21	10.03	0.37	RF

Note : Bold values represent the highest correlation coefficient for DBP and SBP.

**Figure 3.**
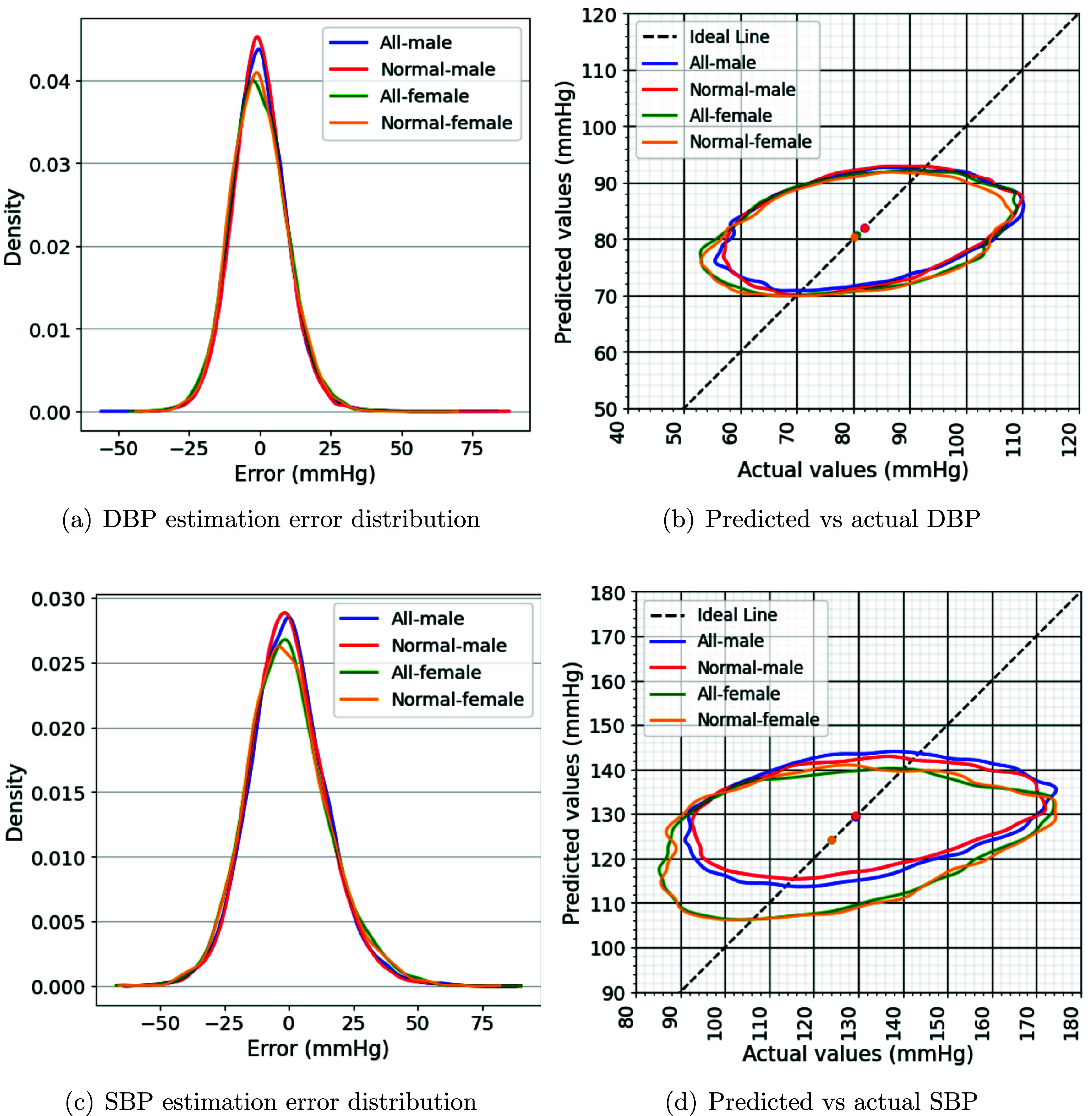
Performance comparison of regression models for estimating systolic (SBP) and diastolic (DBP) blood pressure from 30 s ECGs using 280 features, grouped by ECG label and sex. (a) and (c) Show error PDFs; (b) and (d), 95% percentile contour plots of predicted vs. actual BP. Dots indicate mean actual and predicted values. For ideal regression, contour plots would be narrow and aligned around the identity line.

Figure 3 illustrates the results of the prediction errors distribution (the difference between predicted and actual BP values) and 95% percentile contour plots of predicted vs. actual BP for the best-performing estimation models. In an unbiased and well-performing model, the predicted BP values should closely match the actual value, and the prediction errors should have a mean of zero—ideally exhibiting a symmetric unbiased distribution around this mean. However, in figure 3, we can see a non-zero mean and skewed error distribution, indicating a systematic bias and asymmetric error.

The performance of the proposed models was assessed using two widely recognized BP evaluation standards: the AAMI and the British Hypertension Society (BHS) (Stergiou *et al* 2018). According to the AAMI standard, a valid BP measurement model must achieve a ME of $\unicode{x2A7D}$5 mmHg and a SD of ME $\unicode{x2A7D}$8 mmHg. The BHS standard, in contrast, grades BP measurement devices based on the cumulative percentage of predictions within 5, 10, and 15 mmHg, assigning Grades A, B, or C accordingly. Based on these criteria, neither the SBP nor the DBP prediction models we developed on our dataset satisfied the AAMI requirements, as both exhibited ME and SD values exceeding the thresholds. With respect to the BHS grading, the SBP model failed to meet the standard, while the DBP model fulfills Grade C performance (table 5).

**Table 5.** Performance of machine learning models (random forest (RF), CatBoost, and light gradient boosting machine (LightGBM)) for estimating systolic (SBP) and diastolic (DBP) blood pressure using 30 s ECGs with a 280-feature set, split by ECG labels and subject sex based on the BHS standard (Stergiou *et al* 2018).

				Cumulative mean absolute error			
				Percentage			
BP	Label	Sex	Model	$\unicode{x2A7D}$5 mmHg	$\unicode{x2A7D}$10 mmHg	$\unicode{x2A7D}$15 mmHg	# Subjects
DBP	All	Male	LightGBM	44.5%	73.9%	89.4%	5236
DBP	All	Female	RF	42.2%	71.4%	88.0%	2176
DBP	Normal	Male	CatBoost	46.2%	74.7%	89.9%	4982
DBP	Normal	Female	RF	42.4%	71.5%	88.4%	2078

SBP	All	Male	LightGBM	30.4%	53.8%	71.5%	5236
SBP	All	Female	RF	28.6%	50.6%	68.8%	2176
SBP	Normal	Male	RF	30.7%	54.1%	72.2%	4982
SBP	Normal	Female	RF	28.5%	50.6%	68.7%	2078

Grade A	—	—	—	60%	85%	95%	≥85
Grade B	—	—	—	50%	75%	90%	≥85
Grade C	—	—	—	40%	65%	85%	≥85

### 3.2. Subject-wise vs. record-wise model training

To evaluate the effect of data partitioning strategies on model performance, we conducted an additional experiment using record-wise cross-validation—where training and validation data were randomly split across individual records, regardless of subject identity (table 6). In this setting, the models achieved significantly higher correlation coefficients of 0.59 for SBP and 0.63 for DBP using RF, compared to 0.29 to 0.37 in the subject-wise setup described earlier. This increase in performance suggests that when data splitting is not performed correctly (i.e. using record-wise instead of subject-wise splitting), the models may be leveraging subject-specific patterns seen during training, rather than learning generalizable physiological relationships between ECG and BP that would transfer to unseen subjects.

**Table 6.** Performance of regression models (random forest (RF), CatBoost, and light gradient boosting machine (LightGBM)) for estimating systolic (SBP) and diastolic (DBP) blood pressure using 30 s ECGs with a 280-feature set, split by ECG labels and subject sex. The models were trained and validated using **record-wise cross validation**, where data from the same subject could appear in both the training and validation sets, while the records are still unique to each set. This partitioning may result in potential subject-level information leakage between training and validation sets (compare and contrast with the subject-wise dataset partitioning results in table 4, which does not have this issue). Additionally, all available records per subject were used, which may have introduced bias due to overrepresentation of certain individuals.

Data				SBP (mmHg)						DBP (mmHg)					
Label	Sex	# Subjects	# Test-records	MAE	STD	ME	STD	*ρ*	Model	MAE	STD	ME	STD	*ρ*	Model
All	Male	5236	100 830	9.93	8.45	0.11	13.04	0.59	RF	6.23	5.35	0.07	8.21	0.63	RF
All	Female	2176	23 597	10.70	8.92	−0.03	13.93	0.58	CatBoost	6.39	5.50	0.09	8.43	**0.65**	RF
Normal	Male	4982	86 368	9.72	8.25	0.12	12.74	**0.61**	RF	6.07	5.19	0.06	7.99	0.63	RF
Normal	Female	2078	20 751	10.61	8.84	0.01	13.82	0.56	RF	6.30	5.43	0.10	8.31	0.64	RF

Note : Bold values represent the highest correlation coefficient for DBP and SBP.

### 3.3. ECG-based BP classification

We further investigated the relationship between ECG and BP within a classification framework, which generally, depending on the dataset, may be an easier or more difficult task than continuous BP value estimation (see (Hastie 2009), chapters 2 and 3)^4^4The relative difficulty of classification versus regression is generally context-dependent; classification may be easier when class boundaries are well separated, but harder under class overlap or imbalance, whereas regression can be simpler when the underlying mapping is smooth and benefits from continuity of the target function. See (Hastie 2009, Muthukumar *et al* 2021).. According to the American Heart Association, adult BP is diagnostically categorized into four groups: Normal, Elevated, Stage 1 Hypertension, and Stage 2 Hypertension (Reboussin *et al* 2018). Table 7 lists these categories along with the corresponding SBP and DBP ranges for each group.

**Table 7.** Adult blood pressure norms based on the health status (Reboussin *et al* 2018).

Category	SBP (mmHg)		DBP (mmHg)
Normal	$ < $120	AND	$ < $80
Elevated	120–129	AND	$ < $80
Hypertension (Stage 1)	130–139	OR	80–89
Hypertension (Stage 2)	$\unicode{x2A7E}$140	OR	$\unicode{x2A7E}$90

Similar to the continuous SBP/DBP estimation scenarios, four decision tree-based classification models were trained, this time aiming to predict BP categories rather than the continuous BP values. To obtain a relatively balanced dataset, we considered a binary classification task: Normal and Elevated BP were grouped as the *Normal* (more specifically, non-hypertensive) class, and Hypertension Stages 1 and 2 were grouped as the *Hypertensive* class. Separate models were trained for males and females. With this grouping, the ratio of Hypertensive to Normal was 62% to 38% in males and 57% to 42% in females. The models were developed using the same 280 feature set as the regression problem (i.e. 278 ECG-based features, the average time gap between each ECG and its corresponding BP measurement, and the subject’s age). The classifiers were trained and validated using a subject-wise approach, and the number of records per subject was limited to a maximum of six (the median of the number of ECG-BP pairs across the dataset). The best models for males and females was CatBoost. The five-fold cross-validation, based on metrics including accuracy, F1 -score, specificity, positive predictive value (PPV), and negative predictive value (NPV), area under the receiver operating characteristic (AUROC) curve and area under the precision-recall (AUPR) curve showed minimal variation across the folds, demonstrating the consistency and robustness of the results.

**Figure 4.**
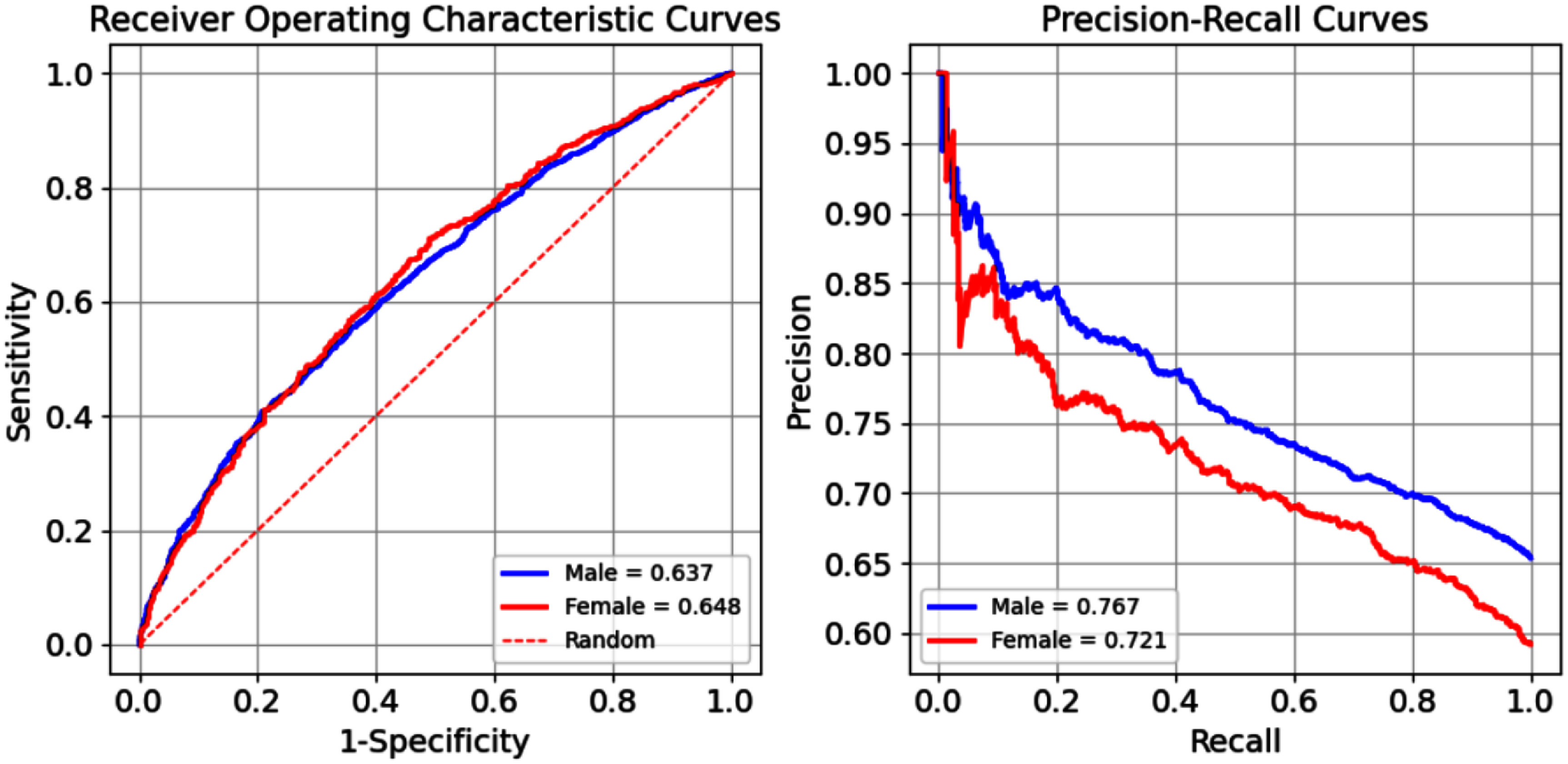
Receiver operating characteristics (ROC) and precision recall (PR) curves of the best-developed classifier (CatBoost) for classifying blood pressure (BP) categories using 30 s ECGs with a 280-feature set, stratified by subject sex. As detailed in the text Normal and Elevated BP categories from table 7 were grouped as *Normal*, and Hypertension Stages 1 and 2 were grouped at *Hypertensive*. Classifiers were trained and validated using subject-wise cross validation, where all data from each subject was assigned exclusively to either the training or validation set. The number of records per subject was limited to a maximum of six to reduce over-representation bias. Differences in the PR curves of male and female datasets are due to varying ratios of *Normal* and *Hypertensive* classes in the male and female datasets.

Table 8 summarizes the results of the developed models by sex and at a sensitivity of 0.7. The best models, Catboost achieved AUROC of 0.636 and 0.655 for male and female datasets, respectively. Figure 4 shows the ROC and PR curves of the models for both groups. The ROC curves of the models exhibit very similar patterns across most sensitivity points. Differences in the PR curves are related to the differences in the ratios of *Normal* and *Hypertensive* classes in the male and female datasets (Sameni 2025). Overall, the classification models show poor performance in prediction of BP groups (*Normal* versus *Hypertensive*).

**Table 8.** Performance of the best-performing classifier (CatBoost) for classifying blood pressure (BP) categories (normal and elevated vs hypertensive cases) using 30 s ECGs with a 280-feature set, per subject sex. The models were trained and validated using leave-subject-out five-fold cross validation, where all data from each subject was assigned exclusively to either the training or validation set to prevent data leakage. The number of records per subject was limited to a maximum of six over-representation bias. The operating point is set to a sensitivity of 0.7. (Abbreviations: PPV: positive predictive value; NPV: negative predictive value; AUROC: area under the receiver operating characteristic curve; AUPR: area under the precision-recall curve).

Sex	AUROC	AUPR	F1-score	Accuracy	Specificity	PPV	NPV
Male	0.636	0.758	0.709	0.624	0.480	0.719	0.457
Female	0.655	0.718	0.693	0.631	0.532	0.686	0.547

## 4. Discussion

This study examined the feasibility of estimating BP using only ECG measurements. Despite leveraging a large and diverse dataset, a comprehensive engineered feature set, and robust ML models, the results suggest that ECG-based BP estimation is not practically viable.

### 4.1. Model performance and generalization

Model performance on the validation set was low, with correlation coefficients around 0.30, indicating poor generalizability.

Apparently, the models consistently predicted BP values centered around the dataset mean. To explore this, we compared the model outputs with fixed values derived from the training set’s mean, median, and mode. As shown in table 9, the ML model predictions were nearly indistinguishable from simply using the median BP value, indicating that the models were effectively regressing to the mean. This phenomenon, known as *central tendency bias* or *regression to the mean* (Barnett 2004), occurs when a model lacks informative input features. In this case, the model appears to ignore ECG variability and rely instead on the statistical distribution of BP in the training data. Formally, this behavior suggests:

\begin{equation*} f\left(\mathrm{BP}|\mathrm{ECG}\right) \approx f\left(\mathrm{BP}\right)\end{equation*}

where $f(\cdot)$ denotes the probability density function, indicating that the ECG features contribute little to the conditional BP distribution.

**Table 9.** Comparison of best-performing machine learning models (random forest (RF), CatBoost, and light gradient boosting machine (LightGBM)) for estimating systolic (SBP) and diastolic (DBP) blood pressure using 30 s ECGs with a 280-feature set, split by ECG labels and subject sex (female (F) and male (M)). Fixed values derived from the training set’s mean, median, and mode are included as constant predictions for comparison. (All values are reported in mmHg). Accordingly, ML-based estimates only marginally outperform fixed estimates using population level priors.

								Fixed BP estimate				Fixed BP estimate				Fixed BP estimate			
				ML BP estimate				(training set mean)				(training set median)				(training set mode)			
BP	Label	Sex	Model	MAE	STD	ME	STD	MAE	STD	ME	STD	MAE	STD	ME	STD	MAE	STD	ME	STD
DBP	All	M	LightGBM	7.62	6.24	0.04	9.85	8.24	6.63	0.04	10.58	8.24	6.63	0.02	10.58	8.30	6.74	−1.17	10.62
DBP	All	F	RF	8.03	6.33	0.19	10.23	8.70	6.77	0.04	11.03	8.68	6.82	−0.50	11.02	9.01	7.21	−3.30	11.06
DBP	Normal	M	CatBoost	7.43	6.08	0.03	9.60	8.08	6.47	−0.01	10.35	8.09	6.49	−0.07	10.37	8.23	6.66	−1.88	10.42
DBP	Normal	F	RF	7.90	6.18	0.21	10.03	8.53	6.62	0.05	10.80	8.52	6.65	−0.35	10.81	8.79	6.93	−1.27	11.12

SBP	All	M	LightGBM	11.82	9.69	0.03	15.29	12.34	10.08	−0.02	15.94	12.33	10.16	−0.96	15.95	12.41	10.28	−2.16	15.97
SBP	All	F	RF	12.59	10.22	0.25	16.21	13.52	10.76	−0.06	17.28	13.49	10.83	−1.02	17.28	13.86	11.38	−3.64	17.56
SBP	Normal	M	RF	11.64	9.49	0.32	15.01	12.22	9.92	0.07	15.73	12.19	9.95	−0.42	15.73	12.29	10.11	−1.83	15.81
SBP	Normal	F	RF	12.56	10.07	0.35	16.09	13.37	10.62	0.01	17.07	13.34	10.69	−0.91	17.07	13.58	10.90	−1.94	17.30

### 4.2. Comparison with prior studies and standards

Our findings support earlier studies, which debated that accurate ECG-based BP estimation is unfeasible (Sato 2021, Landry and Mukkamala 2023), and differ from several earlier studies that reported promising results for ECG-based BP estimation (Mousavi *et al* 2018, Simjanoska *et al* 2018, 2020, Fan *et al* 2020, 2021, Miao *et al* 2020, Banerjee *et al* 2022, Wuerich *et al* 2022, Aldein *et al* 2023, 2025, Syah *et al* 2023, Kuzmanov *et al* 2024), as summarized in table 1. Accordingly, many of these prior studies relied on small sample sizes, lacked subject-level separation between training and testing, or used limited feature sets. The current study, using a large ambulatory dataset and rigorous subject-level validation, offers a more generalizable and conservative evaluation.

To assess the potential for clinical utility, we evaluated our models using two widely accepted standards: the AAMI and BHS. None of the models satisfied the AAMI thresholds (ME $\unicode{x2A7D}$ 5 mmHg, SD $\unicode{x2A7D}$ 8 mmHg), and only the DBP models marginally achieved Grade C according to the BHS scale. These results further emphasize that ECG-only BP estimation does not meet the performance required for clinical use.

### 4.3. Importance of across-subject validation

The subject-wise versus record-wise partitioning of training and validation records is another aspect often undocumented or overlooked in prior studies. Our results show that record-wise validation can significantly inflate model performance by allowing data from the same subject to appear in both training and test sets. This causes subject-level information leakage, particularly when some individuals contribute many records. As a result, the model learns subject-specific patterns rather than general physiological relationships between ECG and BP, performing well on familiar data but failing to generalize to new subjects. In contrast, our subject-wise approach—where all data from each subject was isolated to either training or validation—prevented this leakage and revealed the true complexity of the task. We also limited the number of records per subject to reduce bias from overrepresented individuals. The lower correlation coefficients in this setup provide a more realistic picture of model performance and reflect the actual difficulty of ECG-based BP estimation.

### 4.4. Impact of biological sex on BP

On a population level, males generally have higher average BP values than females (Mousavi *et al* 2024). Our dataset aligns with this result: the mean SBP values were 127.1 mmHg for males and 123.8 mmHg for females, while the mean DBP values were 80.2 mmHg and 78.9 mmHg, respectively. However, the SD of BP values in females was higher than in males, which may be the result of the imbalance number of records between the two groups. Therefore, BP estimation models were developed separately for male and female groups to account for sex-specific physiological differences. Although overall model performance on the validation set was poor (table 4), the correlation coefficient for SBP prediction was higher in females than in males. However, for DBP, the correlation coefficients were very similar across both sex groups. This may be due to the narrower range of DBP values (40–165 mmHg) compared to SBP values (60–227 mmHg). Furthermore, the 95% percentile contour plots (figure 3), which compare predicted versus actual BP values based on sex and ECG labels, indicate that the models predicted a broader prediction range for female subjects, in SBP estimation. This may be due to greater variability or fewer number of records from females in our dataset.

### 4.5. Physiological and statistical implications

From a physiological standpoint, the results can be explained by the fact that BP reflects vascular compliance, peripheral resistance, blood volume, and autonomic tone-factors that are not directly encoded in the electrical activity captured by the ECG. Statistically, this aligns with the concept of *parameter identifiability* in regression problems (Sameni 2023). Even with a large dataset and highly expressive models, some outputs remain fundamentally non-identifiable from a given input modality. Our findings suggest that BP estimation from ECG alone may fall into this category.

### 4.6. Limitations and future work

Several limitations should be acknowledged. First, only single-lead ECGs were used, limiting the available morphological and spatial information. Second, ECG and BP were not recorded simultaneously, though the time gap was constrained to a 3.5 min window and included as a model input. Additionally, all BP values were obtained using non-invasive home devices, which can introduce measurement noise and inaccurate cuff placement and subject positioning. Finally, our dataset may not have fully captured extreme (very low and very high) BP values, which could limit the generalization of our regression and classification models to extreme cases.

Future work should investigate larger BP datasets with full measurement ranges to better capture extremes and assess their impact on model performance. Future work should investigate models that integrate ECG with additional synchronous physiological signals, such as PPG, impedance cardiography, or accelerometry. Multi-lead ECG recordings may also provide more discriminative features. Furthermore, instead of predicting instantaneous BP values, it may be more plausible to estimate average BP over longer time windows. Finally, DL models applied directly to ECG waveforms could be explored, although the identifiability limitation observed in this study may still persist.

## 5. Conclusion

This study critically examined the hypothesis that BP and BP categories can be estimated using only ECG signals. To rigorously test this hypothesis, we developed sex-aware ML regression and classification models using a large ambulatory dataset consisting of 30 s ECG recordings, which are representative of commercially available portable devices. A comprehensive set of 278 engineered ECG features was extracted from the ECGs in addition to key demographic factors (sex and age). Rigorous data pre-processing and subject-level splitting were applied to minimize bias and ensure generalizability.

Despite these comprehensive modeling strategies, the best-performing regression models achieved low correlation coefficients between actual and predicted BP values—0.35 for SBP and 0.38 for DBP—indicating limited predictive power. Moreover, the observed prediction performance was comparable to using simple central tendency measures (e.g. the median BP value) as model outputs. The classification or normal vs hypertensive cases were more promising (with AUROC curve of around 0.64), yet inadequate for reliable ECG-based BP category prediction. These findings suggest that the ECG alone does not carry sufficient information to reliably estimate BP or BP category.

In conclusion, while ECG signals remain highly valuable for a wide range of diagnostic applications, their use in isolation for accurate BP estimation is not feasible based on current evidence. Future research should consider combining ECG with other physiological signals or contextual data to improve BP prediction performance or explore alternative applications where ECG-based modeling may yield more robust results. Furthermore, better results may still be achievable using other methodologies, including DL.

## Data Availability

The data cannot be made publicly available upon publication because they contain commercially sensitive information. The data that support the findings of this study are available upon reasonable request from the authors.

## References

[pmeaae1926bib1] Aldein E A, AbdelRaheem M, Abdellatif M M, Mohamed U S, Atef M (2025). ECG-based blood pressure estimation using a two-stage
inception-regression model.

[pmeaae1926bib2] Aldein E A, Abdleraheem M, Mohamed U S, Atef M (2023). An ECG-based blood pressure estimation using U-Net auto-encoder
and random forest regressor.

[pmeaae1926bib3] Angelaki E (2022). Artificial intelligence-based opportunistic screening for the
detection of arterial hypertension through ECG signals. J. Hypertension.

[pmeaae1926bib4] Angelaki E (2024). Diagnostic performance of single-lead electrocardiograms for
arterial hypertension diagnosis: a machine learning approach. J. Human Hypertension.

[pmeaae1926bib5] Apple
Inc (2025). Hypertension notifications on your apple watch. https://support.apple.com/en-us/117296.

[pmeaae1926bib6] Bahrami Rad A, Galloway C, Treiman D, Xue J, Li Q, Sameni R, Albert D, Clifford G D (2021). Atrial fibrillation detection in outpatient electrocardiogram
monitoring: an algorithmic crowdsourcing approach. PLoS One.

[pmeaae1926bib7] Bahrami Rad A, Kirsch M, Li Q, Xue J, Sameni R, Albert D, Clifford G D (2024). A crowdsourced AI framework for atrial fibrillation detection in
Apple watch and Kardia mobile ECGs. Sensors.

[pmeaae1926bib8] Banerjee S, Kumar B, James A P, Tripathi J N (2022). Blood pressure estimation from ECG data using XGBoost and ANN for
wearable devices.

[pmeaae1926bib9] Barnett A G (2004). Regression to the mean: what it is and how to deal with
it. Int. J. Epidemiol..

[pmeaae1926bib10] Bird K (2020). Assessment of hypertension using clinical electrocardiogram
features: a first-ever review. Front. Med..

[pmeaae1926bib11] Chen Z, Yang X, Teo J T, Ng S H (2013). Noninvasive monitoring of blood pressure using optical
Ballistocardiography and Photoplethysmograph approaches.

[pmeaae1926bib12] Clement D L (2003). Prognostic value of ambulatory blood-pressure recordings in
patients with treated hypertension. New Engl. J. Med..

[pmeaae1926bib13] Clifford G, Azuaje F, McSharry P (2006). Advanced Methods and Tools for ECG Data Analysis (Artech House
Engineering in Medicine and Biology Series).

[pmeaae1926bib14] De Luna A B, Batchvarov V N, Malik M (2006). The morphology of the electrocardiogram. The ESC Textbook of Cardiovascular Medicine Blackwell
Publishing.

[pmeaae1926bib15] Fan X, Wang H, Xu F, Zhao Y, Tsui K-L (2020). Homecare-oriented intelligent long-term monitoring of blood
pressure using electrocardiogram signals. IEEE Trans. Ind. Inf..

[pmeaae1926bib16] Fan X, Wang H, Zhao Y, Li Y, Tsui K L (2021). An adaptive weight learning-based multitask deep network for
continuous blood pressure estimation using electrocardiogram
signals. Sensors.

[pmeaae1926bib17] Fuadah Y N, Lim K M (2022). Optimal classification of atrial fibrillation and congestive
heart failure using machine learning. Front. Physiol..

[pmeaae1926bib18] Graham S L, Drance S M, Wijsman K, Douglas G R, Mikelberg F S (1995). Ambulatory blood pressure monitoring in glaucoma. Ophthalmology.

[pmeaae1926bib19] Hassan M K B A, Mashor M Y, Mohd Nasir N F, Mohamed S (2008). Measuring of systolic blood pressure based on heart
rate.

[pmeaae1926bib20] Hastie T (2009). The elements of statistical learning: data mining, inference, and
prediction.

[pmeaae1926bib21] Huynh T H, Jafari R, Chung W-Y (2019). Noninvasive cuffless blood pressure estimation using pulse
transit time and impedance plethysmography. IEEE Trans. Biomed. Eng..

[pmeaae1926bib22] Johnson A E, Pollard T J, Shen L, Lehman L-w H, Feng M, Ghassemi M, Moody B, Szolovits P, Anthony Celi L, Mark R G (2016). MIMIC-III, a freely accessible critical care
database. Sci. Data.

[pmeaae1926bib23] Kaplan Berkaya S, Uysal A K, Sora Gunal E, Ergin S, Gunal S, Gulmezoglu M B (2018). A survey on ECG analysis. Biomed. Signal Process. Control.

[pmeaae1926bib24] Kario K, Pickering T G, Umeda Y, Hoshide S, Hoshide Y, Morinari M, Murata M, Kuroda T, Schwartz J E, Shimada K (2003). Morning surge in blood pressure as a predictor of silent and
clinical cerebrovascular disease in elderly hypertensives: a prospective
study. Circulation.

[pmeaae1926bib25] Kim C-S, Carek A M, Inan O T, Mukkamala R, Hahn J-O (2018). Ballistocardiogram-based approach to cuffless blood pressure
monitoring: proof of concept and potential challenges. IEEE Trans. Biomed. Eng..

[pmeaae1926bib26] Koscova Z (2024). From sleep patterns to heart rhythms: predicting atrial
fibrillation from overnight polysomnograms. J. Electrocardiol..

[pmeaae1926bib27] Kuzmanov I, Zdravevski E, Lamenski P, Stojkoska B, Bogdanova A M (2024). A study on appropriate segment length for generalized cuff-less
blood pressure estimation from ECG features.

[pmeaae1926bib28] Landry C, Mukkamala R (2023). Current evidence suggests that estimating blood pressure from
convenient ECG waveforms alone is not viable. J. Electrocardiol..

[pmeaae1926bib29] Lee S-S, Nam D-H, Hong Y-S, Lee W-B, Son I-H, Kim K-H, Choi J-G (2011). Measurement of blood pressure using an arterial pulsimeter
equipped with a hall device. Sensors.

[pmeaae1926bib30] Leski J (2002). Robust weighted averaging [of biomedical signals]. IEEE Trans. Biomed. Eng..

[pmeaae1926bib31] Liang C, Yang F, Huang X, Zhang L, Wang Y (2024). Deep learning assists early-detection of hypertension-mediated
heart change on ECG signals. Hypertension Res..

[pmeaae1926bib32] Liu S-H, Cheng D-C, Su C-H (2017). A cuffless blood pressure measurement based on the impedance
plethysmography technique. Sensors.

[pmeaae1926bib33] Luo S, Michler K, Johnston P, Macfarlane P W (2004). A comparison of commonly used QT correction formulae: the effect
of heart rate on the QTc of normal ECGs. J. Electrocardiol..

[pmeaae1926bib34] Ma C, Zhang P, Song F, Sun Y, Fan G, Zhang T, Feng Y, Zhang G (2023). KD-Informer: a cuff-less continuous blood pressure waveform
estimation approach based on single photoplethysmography. IEEE J. Biomed. Health Inf..

[pmeaae1926bib35] Mancia G (2012). Short- and long-term blood pressure variability: present and
future. Hypertension.

[pmeaae1926bib36] Mancia G, Ferrari A, Gregorini L, Parati G, Pomidossi G, Bertinieri G, Grassi G, di Rienzo M, Pedotti A, Zanchetti A (1983). Blood pressure and heart rate variabilities in normotensive and
hypertensive human beings. Circ. Res..

[pmeaae1926bib37] Martin Bland J, Altman D (1986). Statistical methods for assessing agreement between two methods
of clinical measurement. Lancet.

[pmeaae1926bib38] Miao F, Wen B, Hu Z, Fortino G, Wang X-P, Liu Z-D, Tang M, Li Y (2020). Continuous blood pressure measurement from one-channel
electrocardiogram signal using deep-learning techniques. Artif. Intell. Med..

[pmeaae1926bib39] Mousavi S S, Charmi M, Firouzmand M, Hemmati M, Moghadam M, Ghorbani Y (2020). ECG-based blood pressure estimation using mechano-electric
coupling concept. https://arxiv.org/abs/2008.10099.

[pmeaae1926bib40] Mousavi S S, Firouzmand M, Charmi M, Hemmati M, Moghadam M, Ghorbani Y (2019). Blood pressure estimation from appropriate and inappropriate PPG
signals using a whole-based method. Biomed. Signal Process. Control.

[pmeaae1926bib41] Mousavi S S, Guo Y, Robichaux C, Sarker A, Sameni R (2024). Learning from two decades of blood pressure data:
demography-specific patterns across 75 million patient
encounters.

[pmeaae1926bib42] Mousavi S S, Hemmati M, Charmi M, Moghadam M, Firouzmand M, Ghorbani Y (2018). Cuff-less blood pressure estimation using only the ECG signal in
frequency domain.

[pmeaae1926bib43] Mousavi S S, Reyna M A, Clifford G D, Sameni R (2024). A survey on blood pressure measurement technologies: addressing
potential sources of bias. Sensors.

[pmeaae1926bib44] Mukkamala R, Shroff S G, Kyriakoulis K G, Avolio A P, Stergiou G S (2025). Cuffless blood pressure measurement: where do we actually
stand?. Hypertension.

[pmeaae1926bib45] Munoz M L (2015). Validity of (ultra-)short recordings for heart rate variability
measurements. PLoS One.

[pmeaae1926bib46] Muntner P (2019). Blood pressure assessment in adults in clinical practice and
clinic-based research. J. Am. College Cardiol..

[pmeaae1926bib47] Muthukumar V, Narang A, Subramanian V, Belkin M, Hsu D, Sahai A (2021). Classification vs regression in overparameterized regimes: does
the loss function matter?. J. Mach. Learn. Res..

[pmeaae1926bib48] Muzammil M A, Javid S, Afridi A K, Siddineni R, Shahabi M, Haseeb M, Fariha F, Kumar S, Zaveri S, Nashwan A J (2024). Artificial intelligence-enhanced electrocardiography for accurate
diagnosis and management of cardiovascular diseases. J. Electrocardiol..

[pmeaae1926bib49] Nam D-H, Lee W-B, Hong Y-S, Lee S-S (2013). Measurement of spatial pulse wave velocity by using a clip-type
pulsimeter equipped with a Hall sensor and
photoplethysmography. Sensors.

[pmeaae1926bib50] Nateghi M, Sameni R (2025). Estimation-theoretic bias reduction for oscillometric blood
pressure readings. https://arxiv.org/abs/2508.15687.

[pmeaae1926bib51] Neri L (2023). Electrocardiogram monitoring wearable devices and
artificial-intelligence-enabled diagnostic capabilities: a
review. Sensors.

[pmeaae1926bib52] Nichols W W, O’Rourke M, Edelman E R, Vlachopoulos C (2022). Mcdonald’s Blood Flow in Arteries: Theoretical, Experimental and
Clinical Principles.

[pmeaae1926bib53] Okamoto L E, Gamboa A, Shibao C, Black B K, Diedrich A, Raj S R, Robertson D, Biaggioni I (2009). Nocturnal blood pressure dipping in the hypertension of autonomic
failure. Hypertension.

[pmeaae1926bib54] Pickering T G, Hall J E, Appel L J, Falkner B E, Graves J, Hill M N, Jones D W, Kurtz T, Sheps S G, Roccella E J (2005). Recommendations for blood pressure measurement in humans and
experimental animals: part 1: blood pressure measurement in humans: a
statement for professionals from the subcommittee of professional and public
education of the American Heart Association Council on high blood pressure
research. Hypertension.

[pmeaae1926bib55] Podgorelec V, Kokol P, Stiglic B, Rozman I (2002). Decision trees: an overview and their use in
medicine. J. Med. Syst..

[pmeaae1926bib56] Reboussin D M, Allen N B, Griswold M E, Guallar E, Hong Y, Lackland D T, Miller E P R, Polonsky T, Thompson-Paul A M, Vupputuri S (2018). Systematic review for the 2017
ACC/AHA/AAPA/ABC/ACPM/AGS/APhA/ASH/ASPC/NMA/PCNA guideline for the
prevention, detection, evaluation and management of high blood pressure in
adults: a report of the American College of Cardiology/American Heart
Association task force on clinical practice guidelines. J. Am. College Cardiol..

[pmeaae1926bib57] Sameni R (2006–2025). The open-source electrophysiological toolbox (OSET), version
4.0.. https://github.com/alphanumericslab/OSET.

[pmeaae1926bib58] Sameni R (2023). Beyond convergence: identifiability of machine learning and deep
learning models. https://arxiv.org/abs/2307.11332.

[pmeaae1926bib59] Sameni R (2025). On the geometry of receiver operating characteristic and
precision-recall curves. https://arxiv.org/abs/2504.02169.

[pmeaae1926bib60] Sato T (2021). Commentary: assessment of hypertension using clinical
electrocardiogram features: a first-ever review. Front. Med..

[pmeaae1926bib61] Sayk F, Teckentrup C, Becker C, Heutling D, Wellhöner P, Lehnert H, Dodt C (2010). Effects of selective slow-wave sleep deprivation on nocturnal
blood pressure dipping and daytime blood pressure regulation. Am. J. Physiol. Regul. Integr. Comp. Physiol..

[pmeaae1926bib62] Shah K, Pandya A, Kotwani P, Saha S, Desai C, Tyagi K, Saxena D, Puwar T, Gaidhane S (2021). Cost-effectiveness of portable electrocardiogram for screening
cardiovascular diseases at a primary health center in Ahmedabad district,
India. Front. Public Health.

[pmeaae1926bib63] Simjanoska M, Gjoreski M, Gams M, Madevska Bogdanova A (2018). Non-invasive blood pressure estimation from ECG using machine
learning techniques. Sensors.

[pmeaae1926bib64] Simjanoska M, Kochev S, Tanevski J, Bogdanova A M, Papa G, Eftimov T (2020). Multi-level information fusion for learning a blood pressure
predictive model using sensor data. Inf. Fusion.

[pmeaae1926bib65] Stergiou G S (2018). A universal standard for the validation of blood pressure
measuring devices: Association for the Advancement of Medical
Instrumentation/European Society of Hypertension/International Organization
for Standardization (AAMI/ESH/ISO) collaboration statement. J. Hypertension.

[pmeaae1926bib66] Syah K M, Pramudia K N, Rochmad M (2023). Implementation of artificial neural network for real-time blood
pressure estimation using ECG signal.

[pmeaae1926bib67] Wuerich C, Wichum F, El-Kadri O, Ghantawi K, Grewal N, Wiede C, Seidl K (2022). Blood pressure estimation based on
electrocardiograms. Curr. Directions Biomed. Eng..

[pmeaae1926bib68] Zabihi M, Bahrami Rad A, Katsaggelos A K, Kiranyaz S, Narkilahti S, Gabbouj M (2017). Detection of atrial fibrillation in ECG hand-held devices using a
random forest classifier.

[pmeaae1926bib69] Zhang Y, Li Y, Chen X, Deng N (2016). Mechanism of magnetic pulse wave signal for blood pressure
measurement. J. Biomed. Sci. Eng..

[pmeaae1926bib70] Zheng L, Wang Z, Liang J, Luo S, Tian S (2021). Effective compression and classification of ECG arrhythmia by
singular value decomposition. Biomed. Eng. Adv..

